# Supplementary material for: Fine-Tuning Approach for Segmentation of Gliomas in Brain Magnetic Resonance Images with a Machine Learning Method to Normalize Image Differences among Facilities
Source: Cancers (Basel). 2021 Mar 19;13(6):1415. doi: 10.3390/cancers13061415 (PMC8003655; doi:10.3390/cancers13061415)
Supplement: Supplementary file 1 [file cancers-13-01415-s001.pdf]

cancers

Supplementary materials: Fine-Tuning Approach for Segmentation of Gliomas in Brain Magnetic Resonance Images with a Machine Learning Method to Normalize Image Differences among Facilities

Satoshi Takahashi [1,2,†], Masamichi Takahashi [3,4,*,†], Manabu Kinoshita [5], Mototaka Miyake [6], Risa Kawaguchi [7], Naoki Shinojima [8], Akitake Mukasa [8], Kuniaki Saito [9], Motoo Nagane [9], Ryohei Otani [10,11], Fumi Higuchi [10], Shota Tanaka [12], Nobuhiro Hata [13], Kaoru Tamura [14], Kensuke Tateishi [15], Ryo Nishikawa [16], Hideyuki Arita [5], Masahiro Nonaka [17,21], Takehiro Uda [18], Junya Fukai [19], Yoshiko Okita [20,21], Naohiro Tsuyuguchi [18,22], Yonehiro Kanemura [21,23], Kazuma Kobayashi [1,2], Jun Sese [7,24], Koichi Ichimura [4], Yoshitaka Narita [3] and Ryuji Hamamoto [1,2]

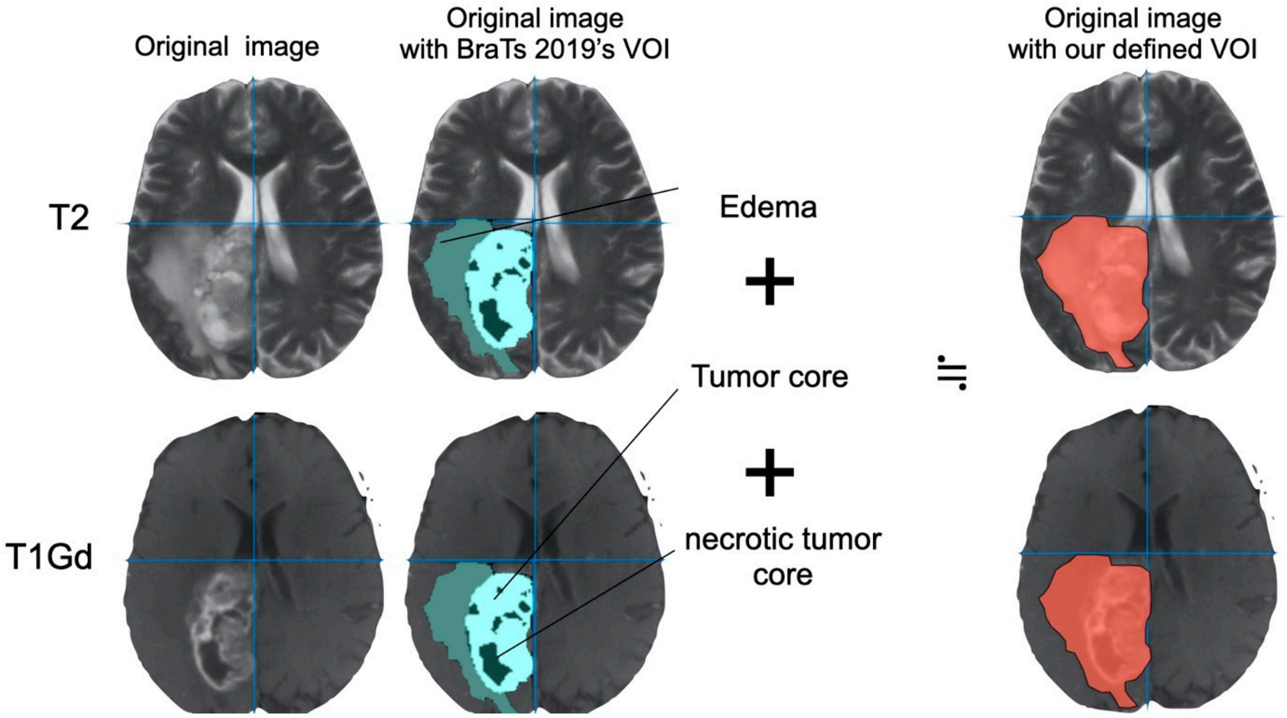

Figure S1. Segmentation results on case 2 from the testing data of JC dataset. The left column is ground truth (made by a skilled radiologist), right is predicted by BraTS model.

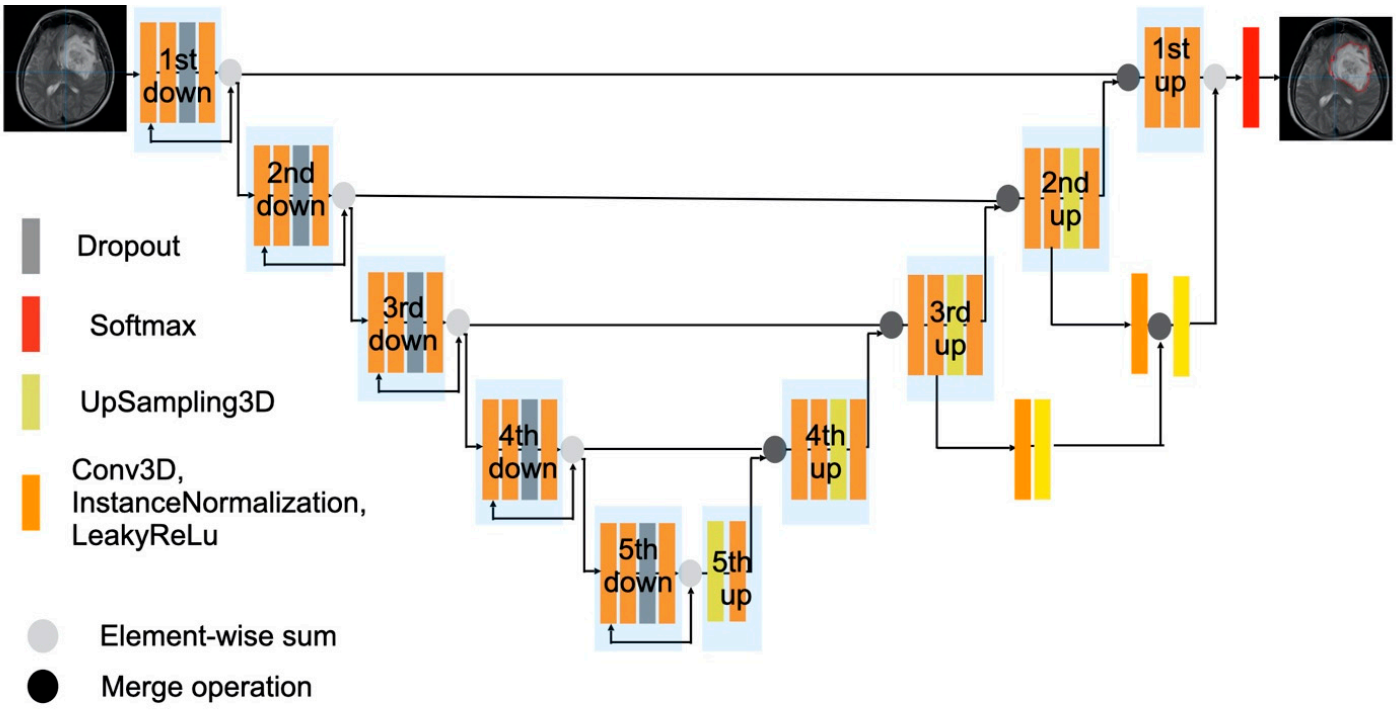

Figure S2. The architecture of machine learning model for segmentation. All layers between two element-wise sums or merged operation layers into one block was grouped. Most blocks contain three 3D convolution layers and one dropout layer. The concept of each group corresponds to the blue shaded areas in **Supplementary Figure S2**. Each block was named in order from the shallowest to the deepest block as '1st down', '2nd down', …'5th down' and then from the deepest to the shallowest block as '1st up', '2nd up',… '5th up'. The optimizer was set to AdaGrad, and the learning rate was set to 0.005.

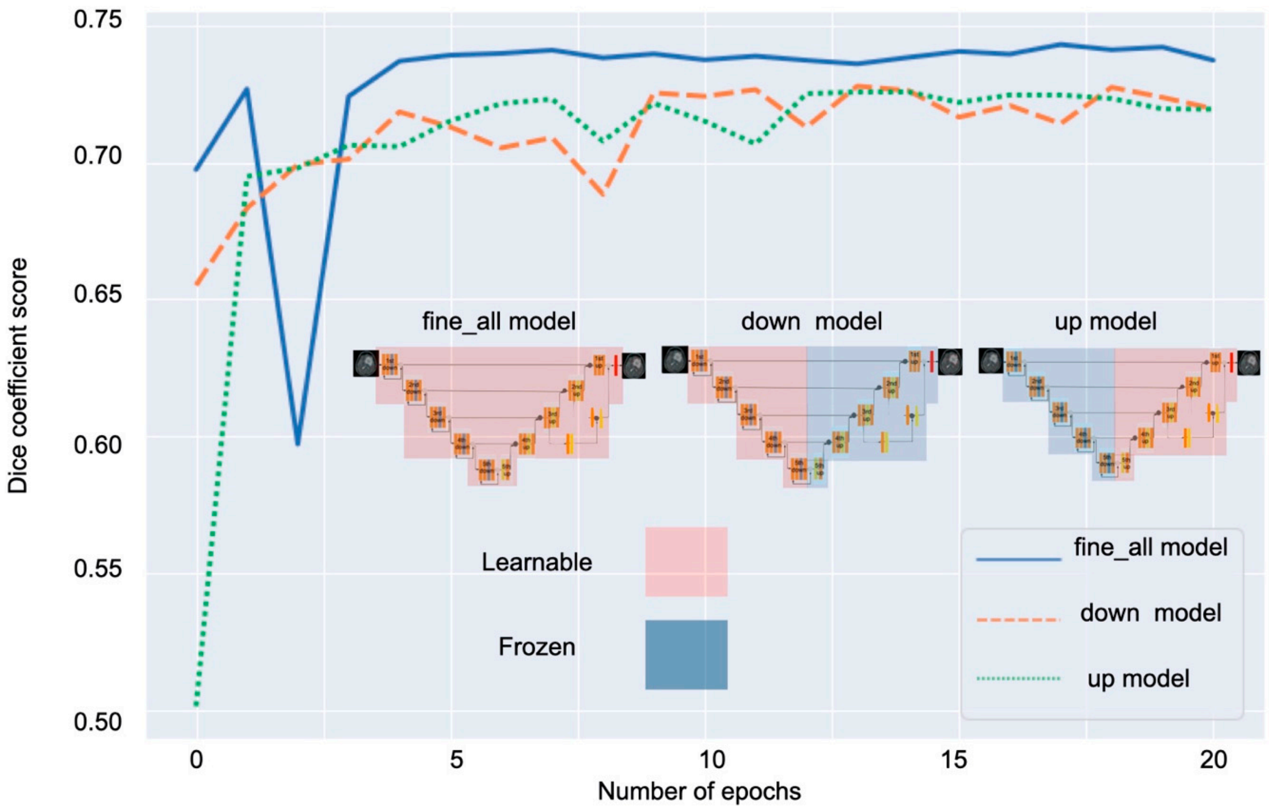

Figure S3. The performances of "fine_all" model, "up" model, and "down" model. The horizontal axis is the number of epochs, and the vertical axis is the Dice coefficient score. The performance of the "fine all" model is better than the performance of others. On the right corner, illustration of fine-tuning method of ""fine_all" method, ""down model" method, and ""up model" method. A red square indicates learnable blocks (group of layers), and a blue square indicates frozen blocks.

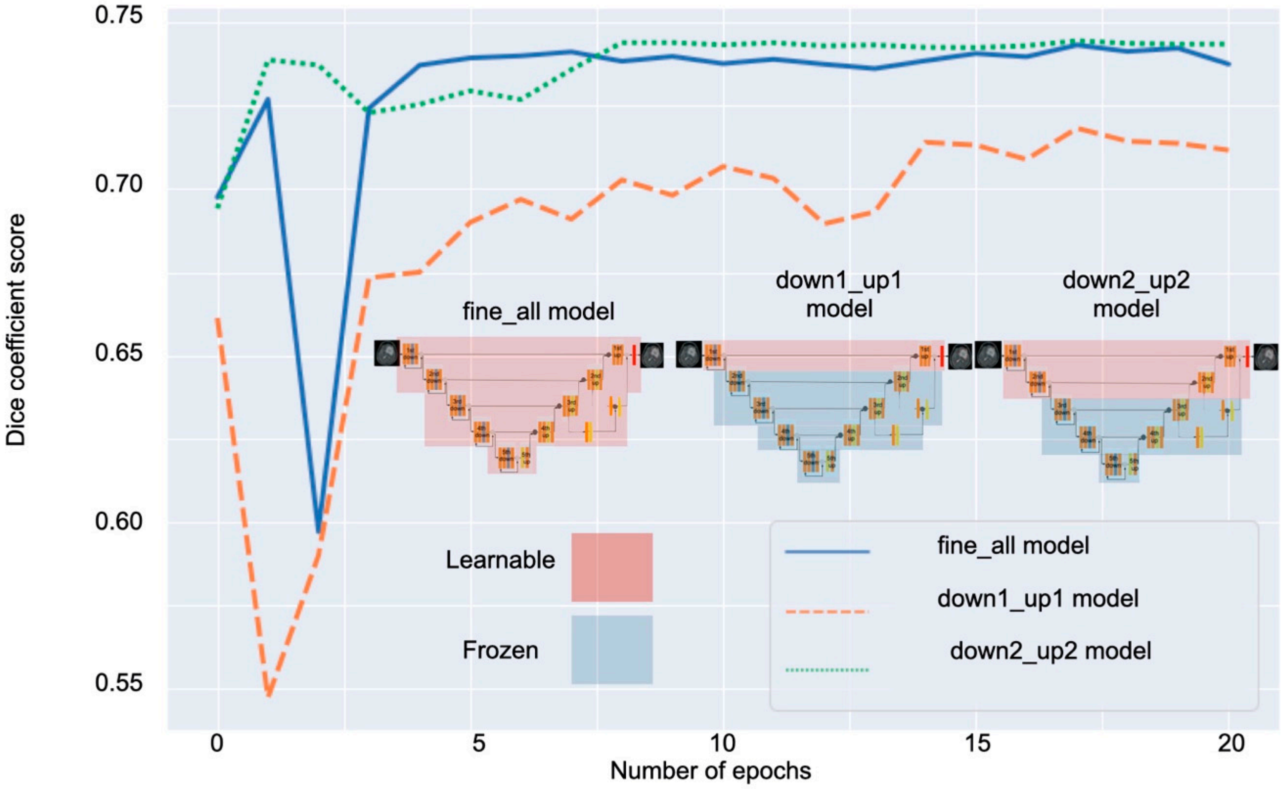

Figure S4. The performances of "fine_all" model, "down1_up1" model, and "down2_up2" model. The horizontal axis is the number of epochs, and the vertical axis is the Dice coefficient score. The performance of "down2_up2" model was almost the same as the performance of the "fine_all" model. On the right corner, illustration of fine-tuning method of ""fine_all model"" method, ""down1_up1 model" method, and ""down2_up2 model" method. A red square indicates learnable blocks (group of layers), and a blue square indicates frozen blocks.

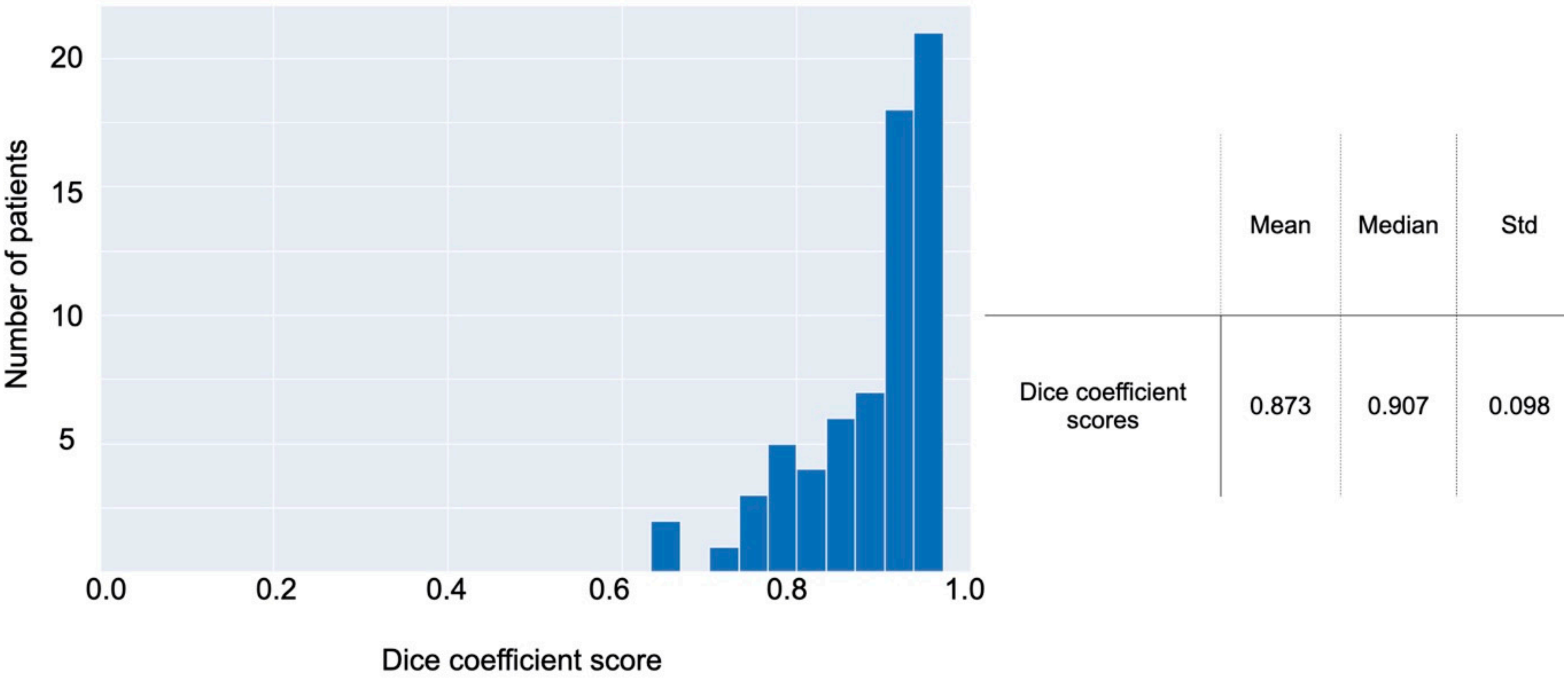

	Mean	Median	Std
Dice coefficient scores	0.873	0.907	0.098

Figure S5. The Dice coefficient scores of the BraTS models for the testing data of the BraTS dataset. On the left is a histogram. The horizontal axis is the Dice coefficient score, and the vertical axis is the number of patients. On the right is a summary of the Dice coefficient score. The Dice coefficient score was high.

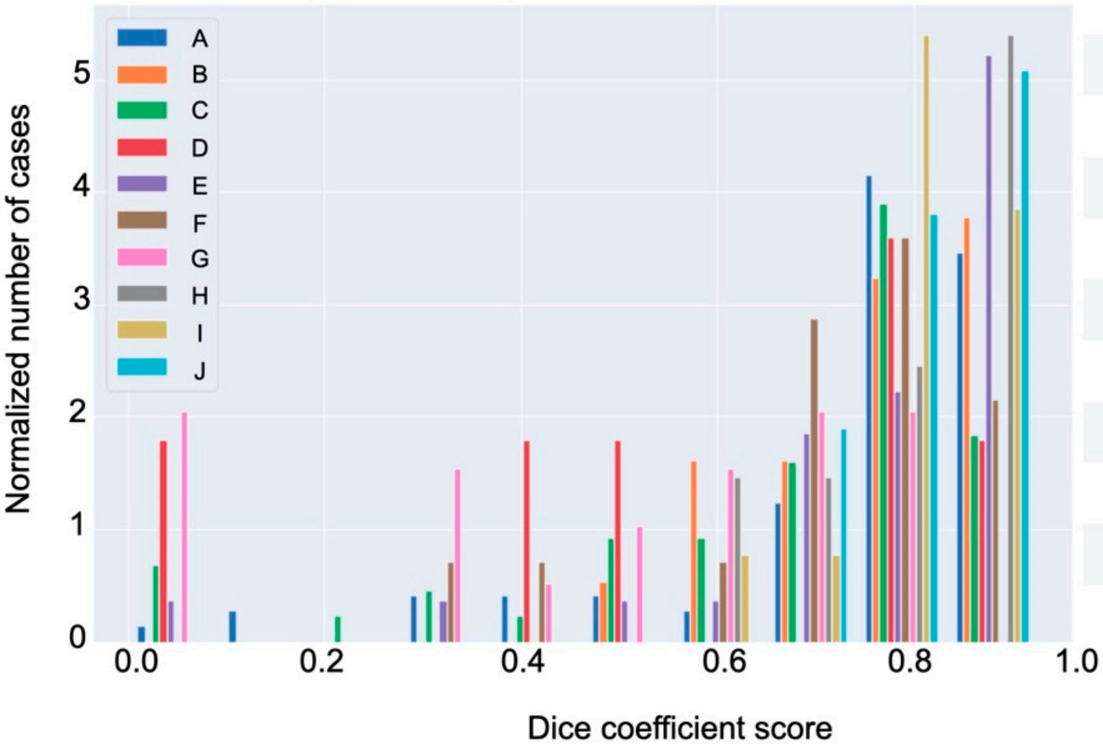

Facility	Mean	Mdedian	Std
A	0.733	0.795	0.196
B	0.768	0.801	0.114
C	0.663	0.755	0.231
D	0.560	0.639	0.318
E	0.766	0.828	0.188
F	0.718	0.745	0.157
G	0.480	0.570	0.286
H	0.798	0.835	0.098
I	0.811	0.801	0.076
J	0.817	0.813	0.065

Figure S6. The Dice coefficient scores of the BraTS model for the testing data of the JC dataset. On the left is a histogram. The horizontal axis is the Dice coefficient score, the vertical axis is the number of cases normalized to form a probability density (the area under each histogram sums to 1), and the colours represent the facilities. On the right is a summary of the Dice coefficient score for the testing data of the JC dataset. The Dice coefficient score significantly decreased from that for the testing data of the BraTS dataset.

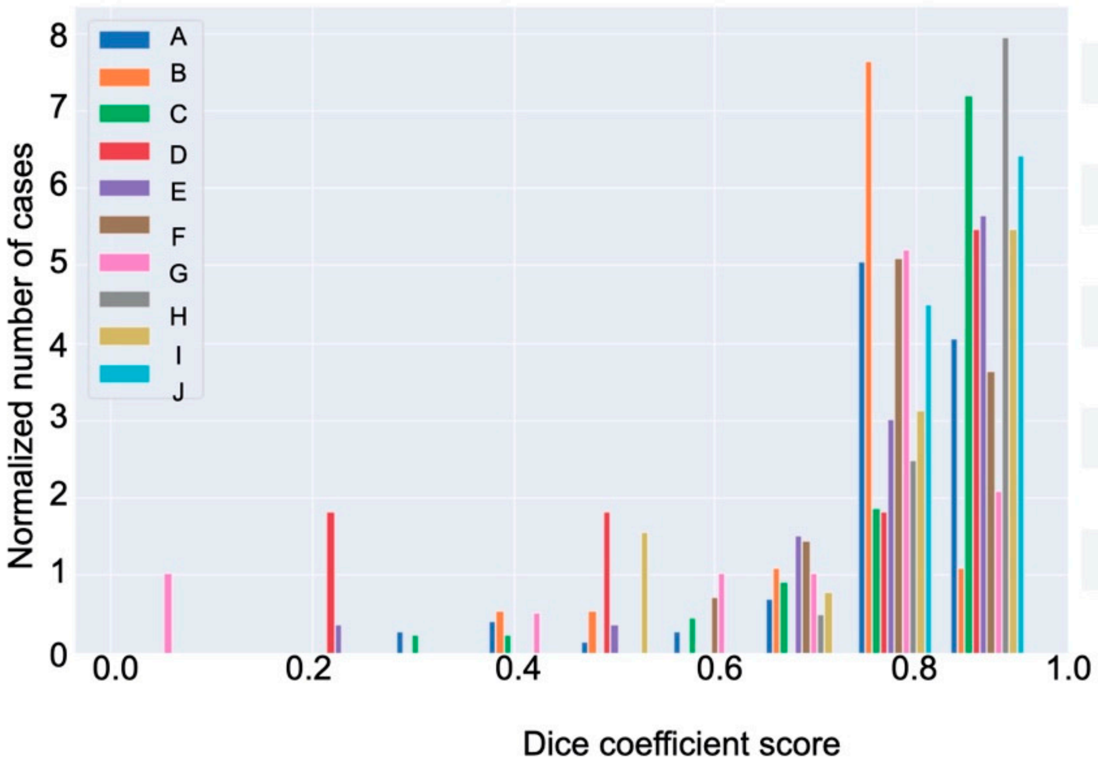

Facility	Mean	Mdedian	Std
A	0.776	0.810	0.133
B	0.740	0.776	0.119
C	0.802	0.845	0.117
D	0.673	0.823	0.273
E	0.785	0.827	0.128
F	0.795	0.805	0.065
G	0.679	0.776	0.238
H	0.836	0.844	0.051
I	0.793	0.827	0.119
J	0.830	0.855	0.052

Figure S7. The Dice coefficient scores of the JC model for the testing data of the JC dataset. The horizontal axis is the Dice coefficient score, the vertical axis is the number of cases normalized to form a probability density (the area under each histogram sum to 1), and the colours represent the facilities. On the right is a summary of the Dice coefficient score for the testing data of the JC dataset. The Dice coefficient score was high.

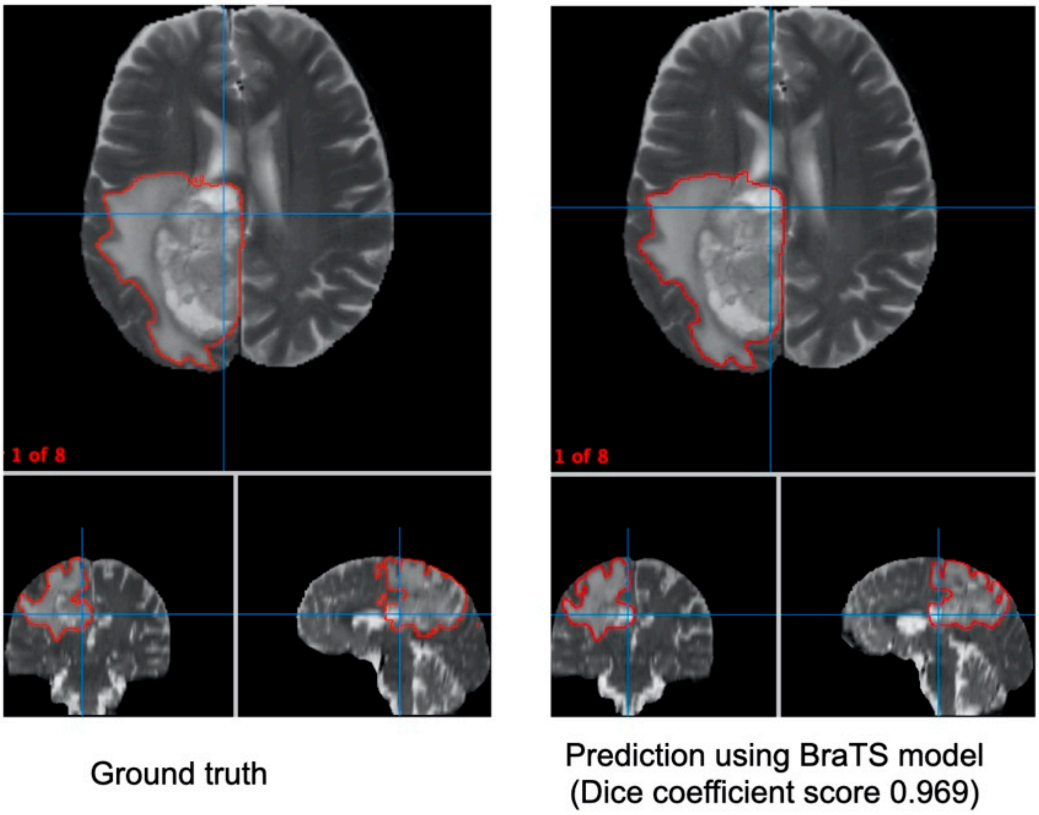

Figure S8. Segmentation results on case 1 from testing data of BraTS dataset. The left column is ground truth, right is predicted by BraTS model.

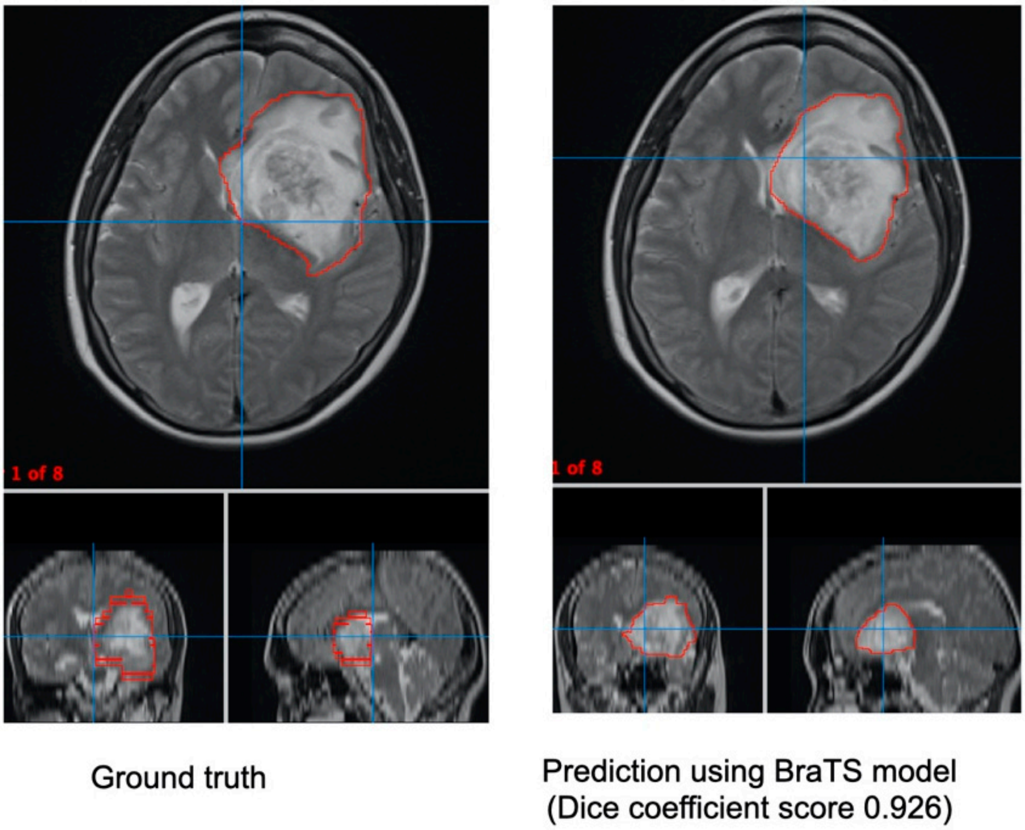

Figure S9. Segmentation results on case 2 from testing data of JC dataset. The left column is ground truth, right is predicted by BraTS model.

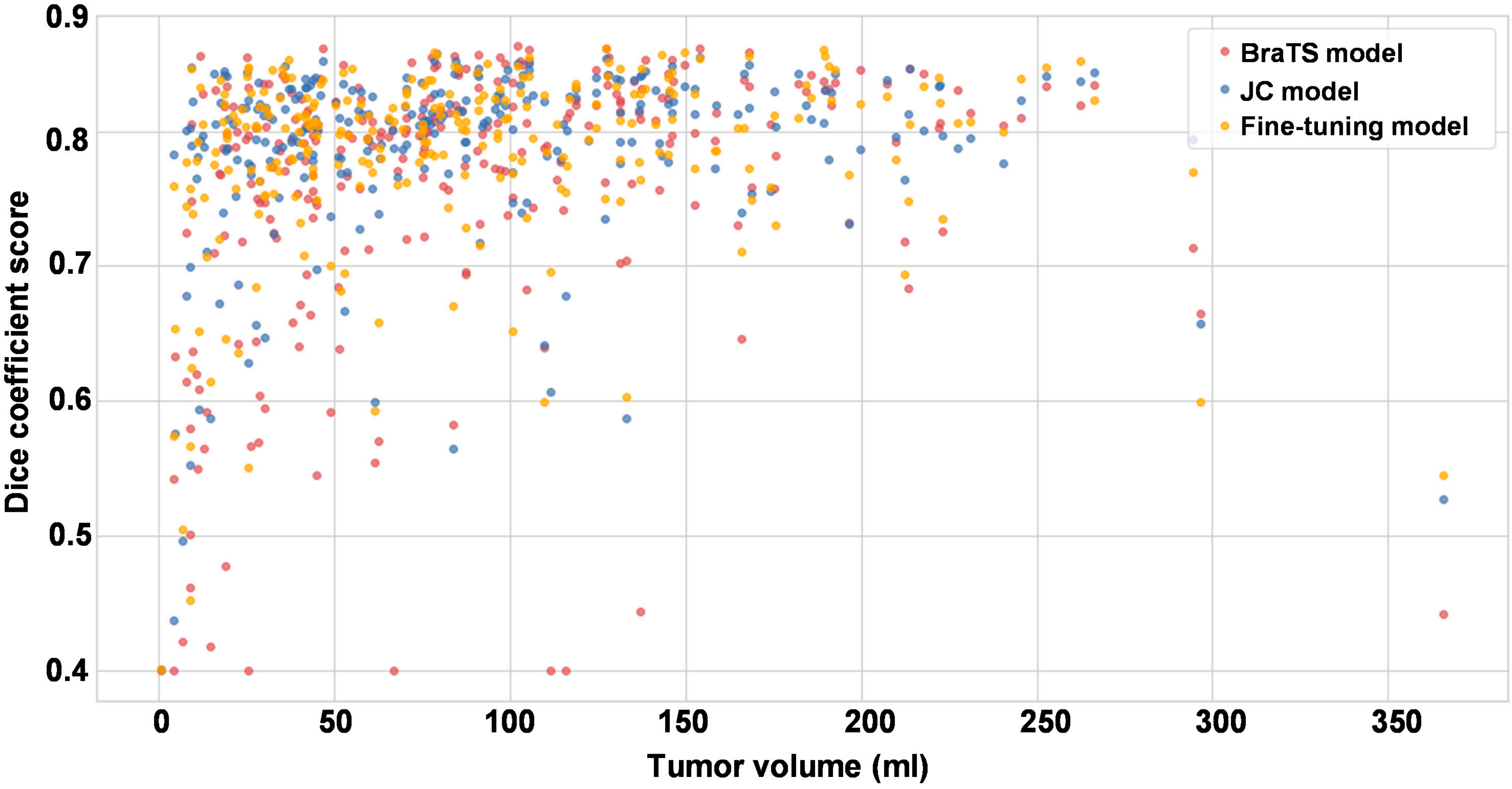

Figure S10. A scatter plots about Dice coefficient score and tumor volumes. The horizontal axis is the tumor volume, the vertical axis is the Dice coefficient score, and the colours indicate the types of machine learning models for segmentation.

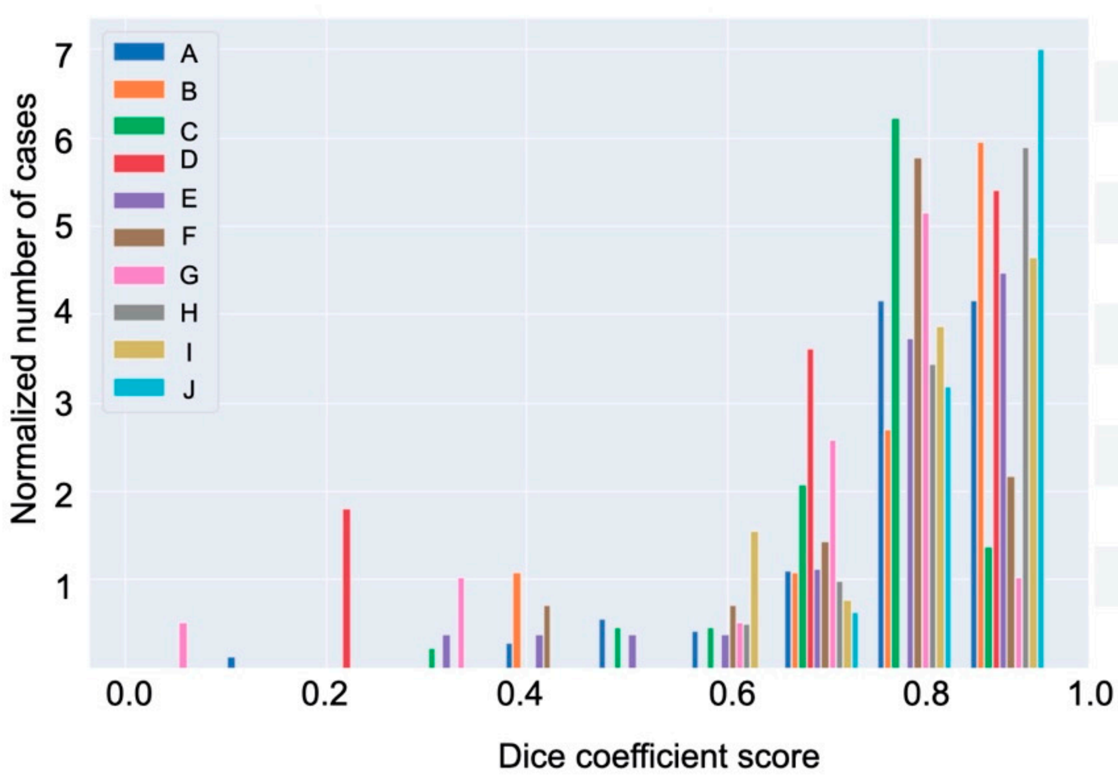

Facility	Mean	Mdedian	Std
A	0.773	0.806	0.139
B	0.794	0.842	0.149
C	0.754	0.797	0.104
D	0.685	0.770	0.244
E	0.764	0.815	0.145
F	0.755	0.784	0.109
G	0.676	0.751	0.211
H	0.830	0.849	0.082
I	0.785	0.815	0.086
J	0.840	0.870	0.070

Figure S11. The Dice coefficient scores of the Fine-tuning models for the testing data of the JC dataset. The horizontal axis is the Dice coefficient score, the vertical axis is the number of cases normalized to form a probability density (the area under each histogram sum to 1), and the colours represent the facilities. On the right is a summary of the Dice coefficient score for the testing data of the JC dataset. The Dice coefficient scores were mostly same as that of the JC model.

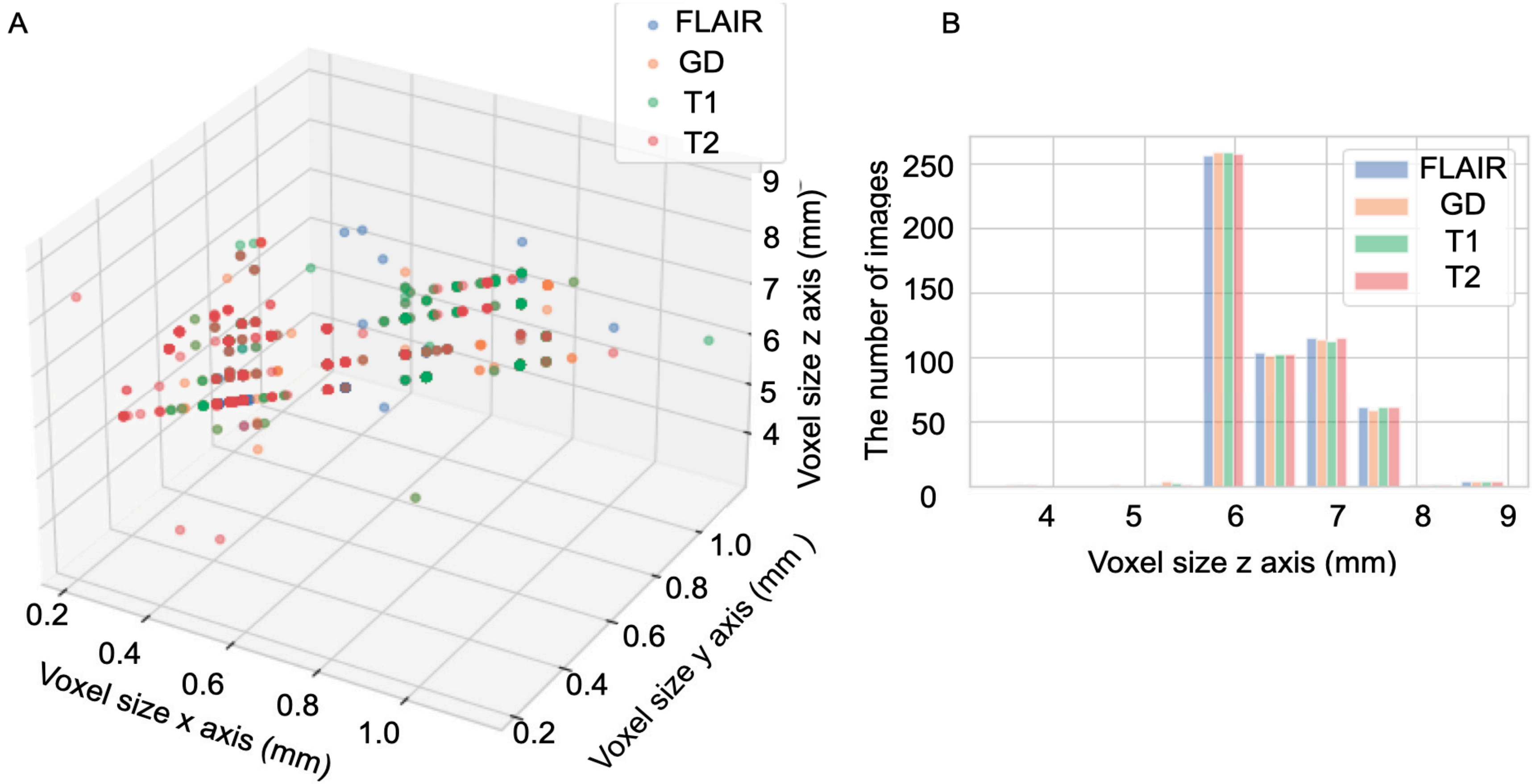

Figure S12. Summary of voxel sizes of images of JC dataset. (**A**) 3D scatter plot of image voxel size. Each axis represents voxel size (mm) and colours means type of image sequences. (**B**) Histogram of z axis voxel size (slice thickness). .

Table S1. Summary of our 3D U-Net architecture.

	Input shape	Output shape	Kernel size of Convolutional layer	Stride of Convolutional layer
1st down	4, 176, 192, 160	12, 176, 192, 160	3,3,3	1,1,1
2nd down	12, 176, 192, 160	24, 88, 96, 80	3,3,3	2,2,2
3rd down	24, 88, 96, 80	48, 44, 48, 40	3,3,3	2,2,2
4th down	48, 44, 48, 40	96, 22, 24, 20	3,3,3	2,2,2
5th down	96, 22, 24, 20	192, 11, 12, 10	3,3,3	2,2,2
5th up	192, 11, 12, 10	96, 22, 24, 20	3,3,3	2,2,2
4th up	192, 22, 24, 20	48, 44, 48, 40	3,3,3	2,2,2
3rd up	96, 44, 48, 40	24, 88, 96, 80	3,3,3	2,2,2
2nd up	48, 88, 96, 80	12, 176, 192, 160	3,3,3	2,2,2
1 st up	24, 176, 192, 160	1, 176, 192, 160	3,3,3	2,2,2

Table S2. Comparison of the Dice coefficient score for the testing data of the JC dataset.

Facility	BraTS model	JC model	Fine-tuning model
Facility A	0.733±0.196	0.776±0.133	0.773±0.139
Facility B	0.768±0.114	0.740±0.119	0.794±0.149
Facility C	0.663±0.231	0.802±0.117	0.754±0.104
Facility D	0.560±0.318	0.673±0.273	0.685±0.244
Facility E	0.766±0.188	0.785±0.128	0.764±0.145
Facility F	0.718±0.157	0.795±0.065	0.755±0.109
Facility G	0.480±0.286	0.679±0.238	0.676±0.211
Facility H	0.798±0.098	0.836±0.051	0.830±0.082
Facility I	0.811±0.076	0.793±0.119	0.785±0.086
Facility J	0.817±0.065	0.830±0.052	0.840±0.07
all	0.717±0.207	0.779±0.137	0.769±0.138

Table S3. Comparison of the Dice coefficient score for the testing data of the JC dataset focus on pathological diagnosis.

	BraTS model	JC model	Fine-tuning model
Diffuse Astrocytoma	0.722±0.207	0.764±0.178	0.75±0.186
Oligodendroglioma	0.675±0.2	0.771±0.097	0.816±0.069
Anaplastic Astrocytoma	0.729±0.202	0.77±0.144	0.763±0.127
Anaplastic Oligodendroglioma	0.772±0.19	0.81±0.054	0.798±0.088
Glioblastoma	0.71±0.212	0.781±0.136	0.769±0.138

Table S4. A summary of the voxel size of the JC dataset. Each column represents a type of image sequence and an axis. For example, 'FLAIR_x' column summarizes the x axis voxel size of FLAIR.

	FLAIR_x	FLAIR_y	FLAIR_z	GD_x	GD_y	GD_z	T1_x	T1_y	T1_z	T2_x	T2_y	T2_z
mean (mm)	0.615	0.615	6.559	0.643	0.643	6.539	0.631	0.631	6.546	0.495	0.495	6.543
std	0.162	0.162	0.647	0.179	0.179	0.66	0.176	0.176	0.656	0.12	0.12	0.669
min (mm)	0.359	0.359	5.5	0.344	0.344	3.6	0.344	0.344	3.6	0.215	0.215	3.3
max (mm)	1	1	9	0.938	0.938	9	1.146	1.146	9	1	1	9
